# Thermal Friction Enhancement in Zwitterionic Monolayers

**DOI:** 10.1021/acs.jpcc.1c09542

**Published:** 2022-02-01

**Authors:** Melisa M. Gianetti, Roberto Guerra, Andrea Vanossi, Michael Urbakh, Nicola Manini

**Affiliations:** † Dipartimento di Fisica, Università degli Studi di Milano, Via Celoria 16, Milano 20133, Italy; ‡ Center for Complexity and Biosystems, Department of Physics, University of Milan, via Celoria 16, Milano 20133, Italy; ¶ CNR-IOM, Consiglio Nazionale delle Ricerche, Istituto Officina dei Materiali, c/o SISSA, Via Bonomea 265, 34136 Trieste, Italy; § International School for Advanced Studies (SISSA), Via Bonomea 265, 34136 Trieste, Italy; ∥ Department of Physical Chemistry, School of Chemistry, The Raymond and Beverly Sackler Faculty of Exact Sciences and The Sackler Center for Computational Molecular and Materials Science, Tel Aviv University, Tel Aviv 6997801, Israel

## Abstract

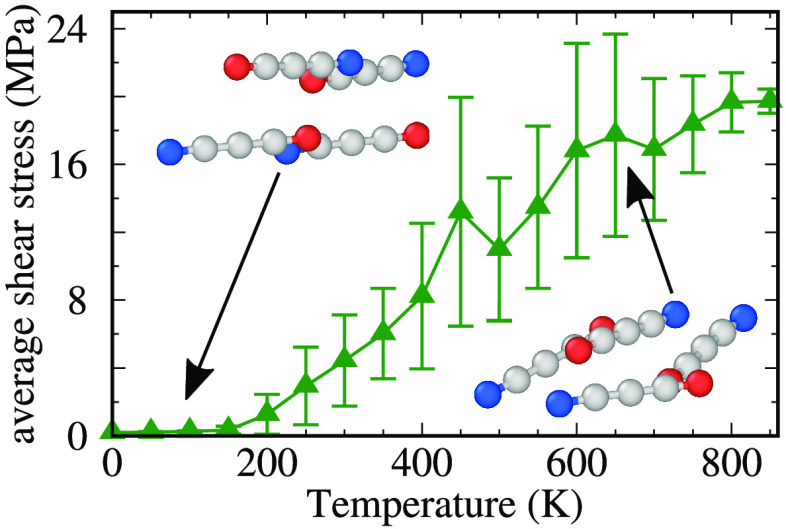

We introduce a model for zwitterionic monolayers and investigate its tribological response to changes in applied load, sliding velocity, and temperature by means of molecular-dynamics simulations. The proposed model exhibits different regimes of motion depending on temperature and sliding velocity. We find a remarkable increase of friction with temperature, which we attribute to the formation and rupture of transient bonds between individual molecules of opposite sliding layers, triggered by the out-of-plane thermal fluctuations of the molecules’ orientations. To highlight the effect of the molecular charges, we compare these results with analogous simulations for the charge-free system. These findings are expected to be relevant to nanoscale rheology and tribology experiments of locally-charged lubricated systems such as, e.g., experiments performed on zwitterionic monolayers, phospholipid micelles, or confined polymeric brushes in a surface force apparatus.

## Introduction

The possibility of controlling nano- and mesoscale friction and mechanical response in a variety of diverse physical systems has been investigated extensively in recent years.^1−4^ In particular, in confined geometries, friction is affected by temperature, usually exhibiting a regular “thermolubric” behavior, with friction decreasing as temperature increases at microscopic scales.^5−9^ The rationale for this standard behavior is random thermal fluctuations assisting the sliding interface in the negotiation of interlocking barriers, thus promoting advancement.

The reverse, namely, friction increasing with temperature, is far less common, although it has been observed in specific situations.^3,10−14^ Certainly, inverted thermolubricity in poor heat-transfer conditions may promote instabilities in the frictional dynamics: the heat dissipated by friction itself can raise temperature, thus triggering a further increase in friction, eventually possibly leading to some kind of lockup, which cuts off this runaway condition.

Inverted thermolubricity was considered primarily as an ingredient for phenomenological models,^15,16^ but it was also investigated in atomic-scale friction within the most basic and fundamental model, namely, the Prandtl-Tomlinson (PT) model,^17^ where it was shown that a peak in friction may arise in a range of temperatures corresponding to a transition from a multiple-slip regime (low *T*) to a single-slip regime (high *T*). However, that simple model fails to reproduce the observed features of the temperature and velocity dependence of friction and of the corresponding force traces measured via atomic-force microscopy (AFM).

Modeling via molecular dynamics (MD) simulations, as a sort of controlled computational “experiment”, has been revealed to be extremely useful in investigating frictional processes of complex systems,^18−20^ possibly avoiding interpretative pitfalls arising from indirect or ex-situ characterization of contact surfaces.

In this work, we investigate the possibility of an inverted thermal dependence of friction between zwitterionic head groups in relative sliding motion. We investigate if, and how, the thermal disordering and rearrangement of such zwitterionic head groups leads to an increase of friction, at least over suitable temperature ranges, especially those experimentally relevant.

We simulate zwitterionic molecules, flexible linear macromolecules that can be tethered to a surface with the aim of modifying its distinctive properties. These molecules can have charge-free or zwitterionic terminations, depending on the specific surface features that are addressed.^21^ Applications involving zwitterionic molecules include colloid stabilization, regulation in wetting and adhesion, and the formation of protective coatings, among many others.^22−24^ Even though vast theoretical research on zwitterionic molecule lubrication has been carried out,^25−27^ there remain unanswered questions about the microscopic mechanisms of friction, especially under the influence of temperature, on surfaces decorated or covered with these complex molecules.

Here, we develop a coarse-grained model to study friction between two preassembled zwitterionic monolayers.^28−30^ Our simulations demonstrate how the modification of the geometric rearrangement of locally zwitterionic molecular portions gives rise to different interlocking configurations at the sliding interface, leading to distinct frictional regimes, as a function of temperature.

## Methods

### The Model

**Figure 1:**
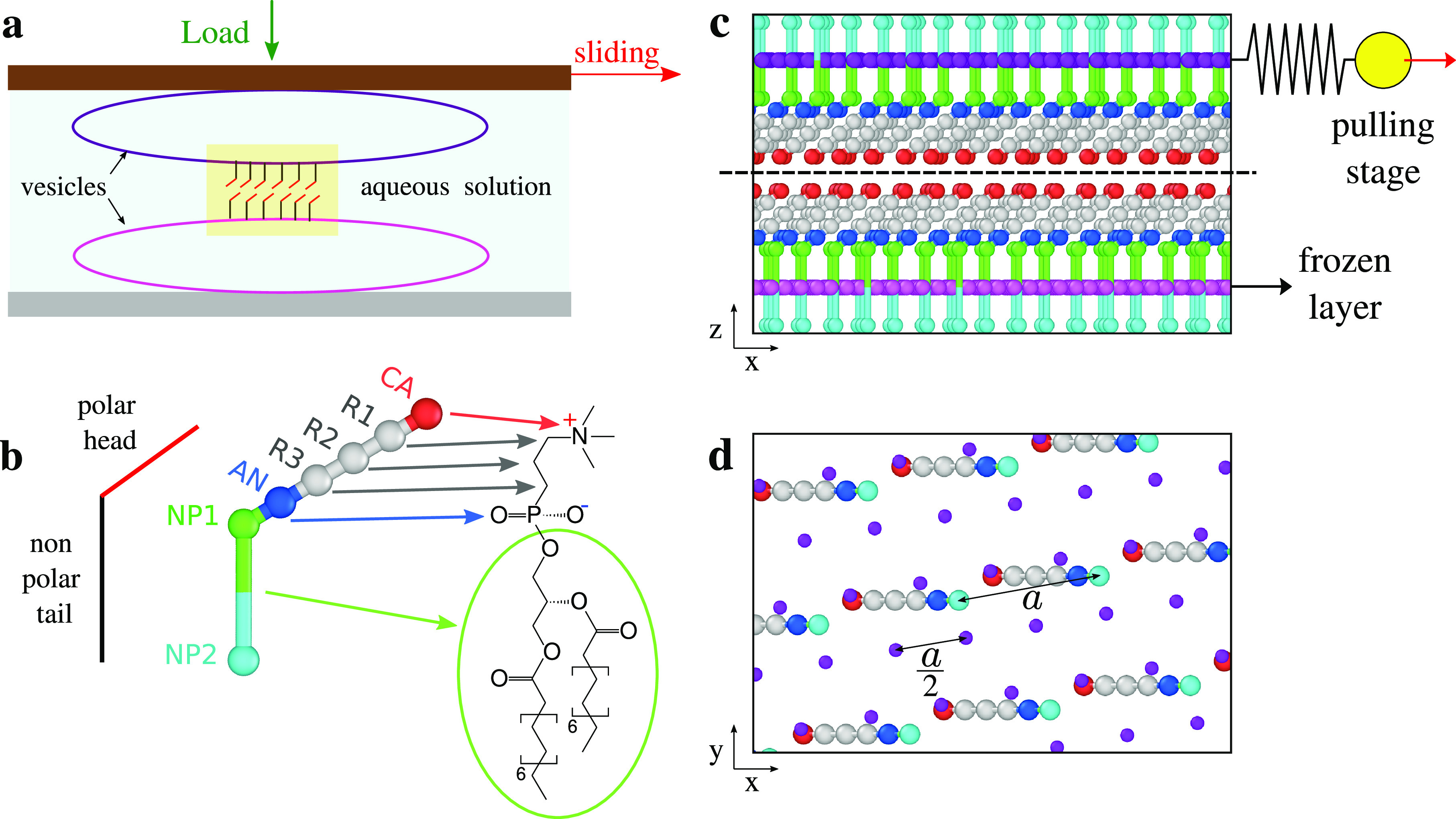
(a) Sketch of the experimental SFA setup. (b) Three representations of one molecular unit in the vesicle wall. Left: a radically simplified sticks scheme, as in panel (a). Center: the simulated united-atom molecule. Right: the detailed chemical structure of a dipalmitoylphosphatidylcholine molecule. (c) The initial regularly spaced configuration of the system (side view). Magenta, purple, red, blue, green, and cyan spheres represent SUB and SUP layers, cations, anions, and NP1 and NP2 particles, respectively. Gray particles represent the R1, R2, and R3 neutral residues in between the cation and the anion. The yellow sphere represents the pulling stage advancing with constant speed. The dashed line marks the sliding interface. (d) Top view restricted to the particles above the black dashed line in (c); purple SUP particles are drawn smaller for better readability.

We propose a model inspired by surface force apparatus (SFA) experiments with confined self-assembled vesicles formed by organic polymers composed of hydrophobic tails and hydrophilic heads consisting of short zwitterionic chains.^31−33^ The self-arranged vesicles can sit inside the SFA confined contact, as sketched in Figure 1a: this arrangement provides a highly sensitive setup for the measurement of the frictional shear stress between well-characterized flat surfaces with molecular layers sticking out of them in mutual shearing motion and under a controlled normal load.^34^ The solid surfaces and the viscoelastic deformability of the vesicles cooperate in generating an essentially atomically flat interface between the exposed surfaces of two contacting vesicles. This flat interface extends over the size of a vesicle, namely, several micrometers across. All shearing occurs in this sliding interface, which is therefore responsible for all observed frictional forces.^31−33,35,36^

A minimal model for simulating the frictional properties of this interface must include at least the exposed zwitterionic head groups of the molecules sticking out from the vesicle, as sketched in Figure 1b. We simulate these heads in a united-atom style as a string of 5 point-like particles. From the interface point of view, the rest of the molecules, namely, the glycerol group and the long alkyl tails have the role of providing a directed support to the heads and transmitting the load and shear forces acted by the SFA setup. In a coarse-grained representation of the hydrophobic inner part of the vesicle, we model this part of the system by parallel rigid layers, one for each contacting vesicle: we name them SUP and SUB layers. The lateral arrangement of molecules in these two layers is modeled using triangular lattices whose periodicity *a*/2 of 0.41 nm sets the equilibrium intermolecular spacing (see Figure 1d). This spacing *a* is the characteristic distance between neighboring molecules, matched to the typical areal number density of these vesicle-forming polymers, namely, 1.72 molecules/nm^2^.^37^ To prevent trivial and unrealistic perfect-commensuration effects, we impose a relative angle of rotation ϕ between the layers’ crystalline directions (opposite rotations by ±ϕ/2 for each layer). The value of ϕ = 19.65° and the numbers of lattice repetitions in the rigid layers are adapted in order to fit a supercell periodic in the horizontal *xy* plane accommodating both lattices, as detailed in the Supporting Information. We represent the experimental mesoscopic interface within a rectangular simulation supercell with dimensions *l*_*x*_ = 8.32 nm and *l*_*y*_ = 14.41 nm. The supercell contains 206 molecules in each layer and 4 times as many atoms in each of the SUP and SUB rigid layers.

The link between the zwitterionic molecular part and the rigid layers is provided by the NP1 and NP2 units of each molecule that represent the nonpolar tails as a pair of point-like particles. Each of these pairs of bonded atoms remains “planted” in one (out of four) of the lattice nodes in either the SUP or the SUB layer. The intralayer–molecule interaction results in keeping these nonpolar sections close to vertical alignment and regularly spaced, while allowing for a limited degree of elastic deformability; see Figure 1b,c.

The SUB-layer particles (magenta particles in Figure 1c) are kept fully frozen. Those in the SUP layer (purple particles in Figure 1c,d) are constrained to form an identical, yet misaligned, rigid layer, allowed to translate in the three directions.

Each embedded macromolecule therefore consists of a chain of seven particles, as depicted in Figure 1b. It starts with a cation (CA, red particle) followed by three uncharged residues (R1–R3, gray), an anion (AN, blue), and two uncharged particles, NP1 (green) and NP2 (cyan). Inspired by the dipalmitoylphosphatidylcholine molecule, we set the masses (in a.m.u.) of the molecular beads to 60 for CA, 15 for R1–R3, 80 for AN, and 50 for NP1 and NP2. The CA and AN beads carry an associated charge of *q* = 0.25 and −*q*, respectively, in elementary-charge units, while all other beads are neutral. For comparison, we also consider a charge-free version of the model with *q* = 0 for all beads. Successive atoms in each chain are connected by elastic springs representing both the stretching and the angular degrees of freedom. All equilibrium angles *θ*_eq_ are 180°, except for the NP2–NP1–AN angle, which we set to 111°, representative of a sp^3^ skeleton oxygen, attempting to keep the zwitterionic heads tilted away from being vertical.^38^

The intramolecular harmonic interactions follow the standard expression
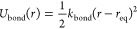
for linear springs, and
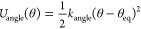
for angular springs. Table 1 lists the parameters adopted for both kinds of intramolecular bonding interaction.

**Table 1:** Intramolecular Interaction Parameters for the Bonded Interactions^a^

Harmonic Bonds			
particle 1	particle 2	*k*_bond_ (N·m^–1^)	*r*_eq_ (nm)
CA	R1	480	0.16
R1	R2		
R2	R3		
R3	AN		
AN	NP1		
NP1	NP2		0.67

Harmonic Angular Interactions				
particle 1	particle 2	particle 3	*k*_angle_ (eV·rad^–2^)	θ_eq_ (degree)
CA	R1	R2	20	180
R1	R2	R3		
R2	R3	AN		
R3	AN	NP1		
AN	NP1	NP2	2	111

a All interactions not listed here involve non-bonded atoms and are of the Morse-type, eq 1.

For the nonbonded pairwise particle–particle interactions, we adopt a Morse potential
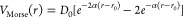
with a standard shift and a linear term added so that both potential energy and force drop to zero at a cutoff distance *R*_c_; see the Supporting Information. The stiffness parameter α = 15 nm^–1^ and the cutoff distance *R*_c_ = 1 nm are the same for all interaction pairs. The values of the potential well depth *D*_0_ and equilibrium spacing *r*_0_ are listed in Table 2. Note that, while all nonbonded beads interact pairwise through Morse terms, as an exception, cross-layer interactions are restricted to the polar heads of the molecules (CA, R1–R3, and AN spheres in Figure 1) with spurious cross-layer terms removed by the *D*_0_ = 0 values in Table 2.

**Table 2:** Values for the Morse Interaction Parameters between Pairs of Particles

particle 1	particle 2	*D*_0_ (eV)	*r*_0_ (nm)
default	default	0.010	0.41
SUP	NP1/2 (SUP layer)	5.0	0.41
SUP	NP1/2 (SUB layer)	0.0	
SUB	NP1/2 (SUB layer)	5.0	
SUB	NP1/2 (SUP layer)	0.0	
SUB	SUP	0.0	
NP1 (SUP layer)	NP1 (SUP layer)	5.0	0.82
NP1 (SUP layer)	NP1 (SUB layer)	0.0	
NP1 (SUB layer)	NP1 (SUB layer)	5.0	
NP2 (SUP layer)	NP2 (SUP layer)	5.0	
NP2 (SUP layer)	NP2 (SUB layer)	0.0	
NP2 (SUB layer)	NP2 (SUB layer)	5.0	

Within the cutoff radius, Coulombic pairwise interactions are computed directly in real space, while outside that distance interactions are evaluated in reciprocal space. For the reciprocal space, a particle–particle particle–mesh solver (PPPM)^39,40^ is used with a precision of 10^–4^ eV·nm^–1^, which proved to be sufficiently accurate; see further details in the Supporting Information.

### Simulations

We adopt LAMMPS^41^ as the simulation platform for integrating the equations of motion
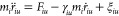
1In addition to the conservative forces *F*_*iu*_ explicitly provided by the force fields described in the previous section, we impose a finite temperature using a Langevin thermostat with a damping rate γ_*iu*_ applied to all particles forming the molecules and Gaussian random forces ξ_*iu*_.^42^ This thermostat is set to act only along the coordinates *u* = *y*, *z* in order to prevent any spurious thermostat-originated frictional damping along the most relevant sliding direction *u* = *x*.^43,44^Figure S1 illustrates the robustness of the friction simulated in our model against the precise value of γ_*iy*_ = γ_*iz*_ = γ adopted. Eventually, we select a value of γ = 1 ps^–1^ for all simulations.

While experiments with this kind of setup are usually carried out in (typical aqueous) solution, here, with the aim of providing a qualitative phenomenology (independent of the specific solvent nature), we adopt a suitably enlarged residue size *r*_0_ and exploit a Langevin approach effectively taking care of degrees of freedom inherent in the real, physical system, which are not explicitly included in our model.^45^ Besides, a real solvent introduces electrostatic screening, which is partly accounted for in this model by the relatively small charges on the AN and CA residues. As we intend to address a general mechanism without focusing on a specific system, we leave out all distance, frequency, and temperature dependence of the screening that a real solvent would entail.

For each simulation, we prepare an initial configuration by executing a sufficiently long “running in” simulation starting from the initial state shown in Figure 1c, letting the dynamics evolve with the appropriate load, temperature, and fixed sliding velocity of the SUP layer until a steady state is reached. In the appropriate steady-state configuration, we attach a pulling stage to the SUP layer (yellow sphere in Figure 1c) through a spring of stiffness *k* = 1 eV·nm^–2^ ≃ 0.16 N·m^–1^, equivalent to a shear stress per unit elongation *k*/(supercell area) = 1.34 × 10^9^ MPa·m^–1^. We carry out the simulations with the stage advancing at constant speed in the *x* direction: *x*_stage_ = *v*_stage_*t*. A default *v*_stage_ = 5 m·s^–1^, in the range of a typical MD approach, is adopted, but we explore other velocities too. In each simulation, the total advancement of the stage amounts to 100 nm. We obtain the instantaneous shear stress from the spring elongation. We start averaging this shear stress when the system enters a steady sliding state until the end of the simulation. This corresponds to one discarding an initial transient of 20–25 nm until at least the first slip event takes place.

We report the averaged shear stress with vertical bars reflecting the root mean squared fluctuations observed along the corresponding friction trace. Large bars indicate stick–slip dynamics, while small bars originate from smooth sliding.

We apply relatively moderate values of loads (*L*) in the 0–20 MPa range, relevant for SFA experiments on organic macromolecules.

## Results

**Figure 2:**
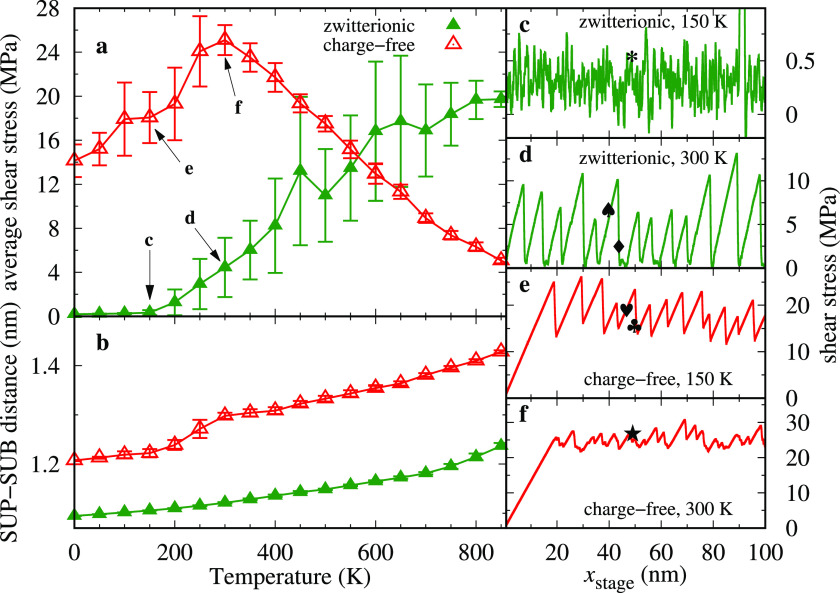
(a) Nonmonotonic variation of the frictional shear stress and (b) the distance between the rigid layers as a function of temperature for zwitterionic and charge-free systems. *v*_stage_ = 5 m·s^–1^, *L* = 10 MPa. (c–f) Shear traces for the pointed temperatures in panel (a). Symbols in (c–f) refer to the snapshots in Figure 3.

**Figure 3:**
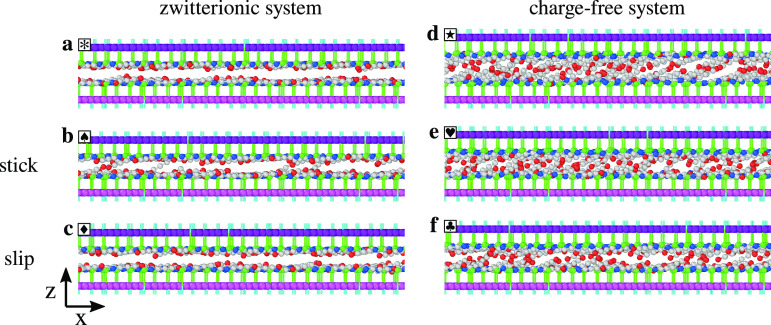
Side views of 5 nm *y*-thick slices of simulation snapshots. Each snapshot corresponds to the time instant marked by the corresponding symbol in panels c–f of Figure 2. (a) Smooth sliding, zwitterionic system, *T* = 150 K, (b) stick point, zwitterionic system, *T* = 300 K, (c) slip point, zwitterionic system, *T* = 300 K, (d) high friction state, charge-free system, *T* = 300 K, (e) stick point, charge-free system, *T* = 150 K, and (f) slip point, system, *T* = 150 K.

**Figure 4:**
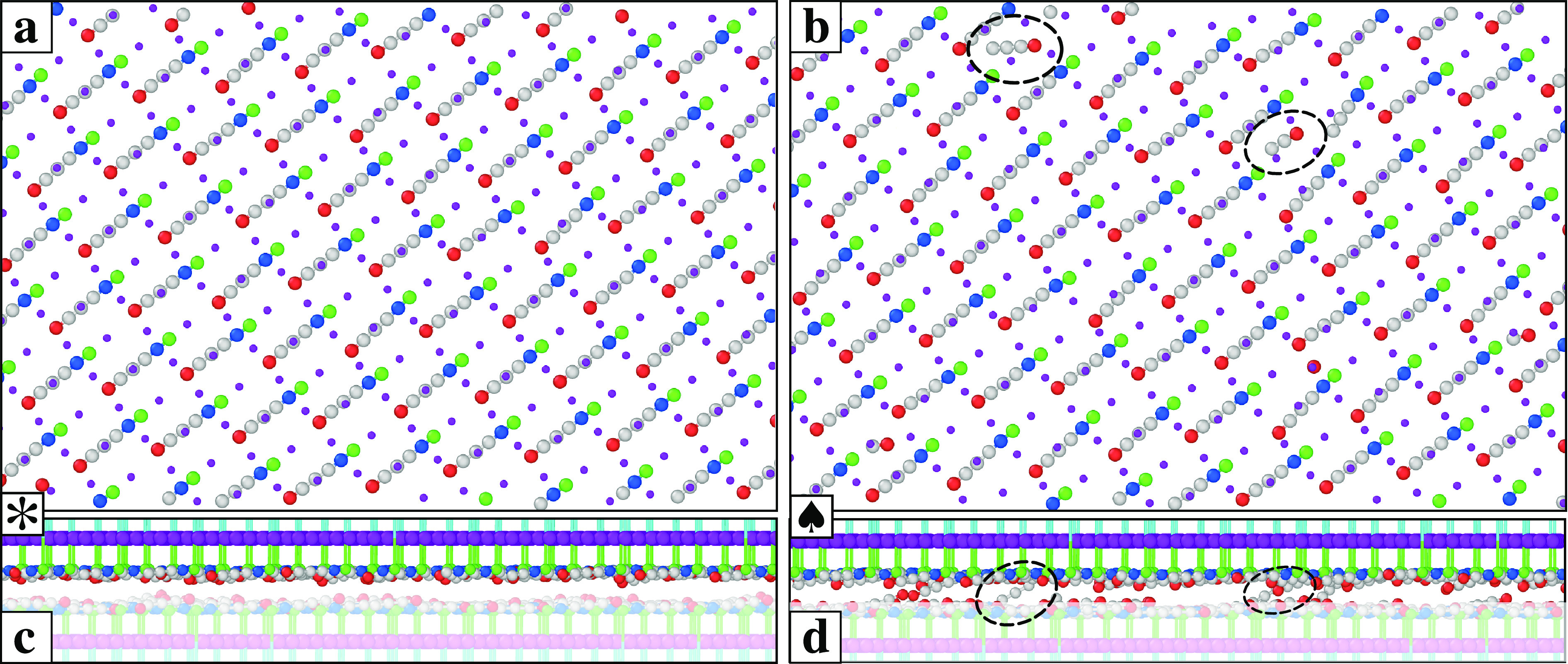
(a, b) Top views of the snapshots of Figure 3a (150 K) and 3b (300 K), including a horizontal slice from the sliding plane in between the chains to immediately above the rigid SUP layer. This slice is the unshaded part of the side views (c) and (d). Black dashed circles highlight SUB chains intersecting the SUP chains’ cation plane. The end cation of each of those SUB chains feels a relatively strong Coulomb interaction with the two top-chain anions, generating the interlocking spots responsible for the stick.

**Figure 5:**
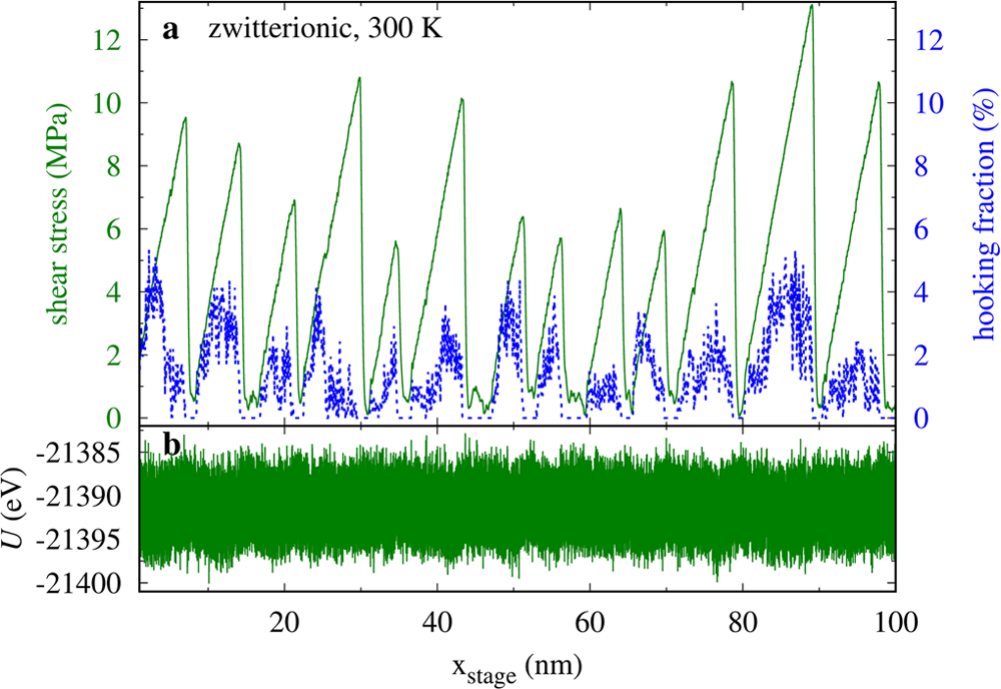
(a) Percentile hooking fraction *h* as a function of the stage displacement correlated with the frictional shear stress for the same simulation as in Figure 2d. (b) The total potential energy *U* for the same simulation.

**Figure 6:**
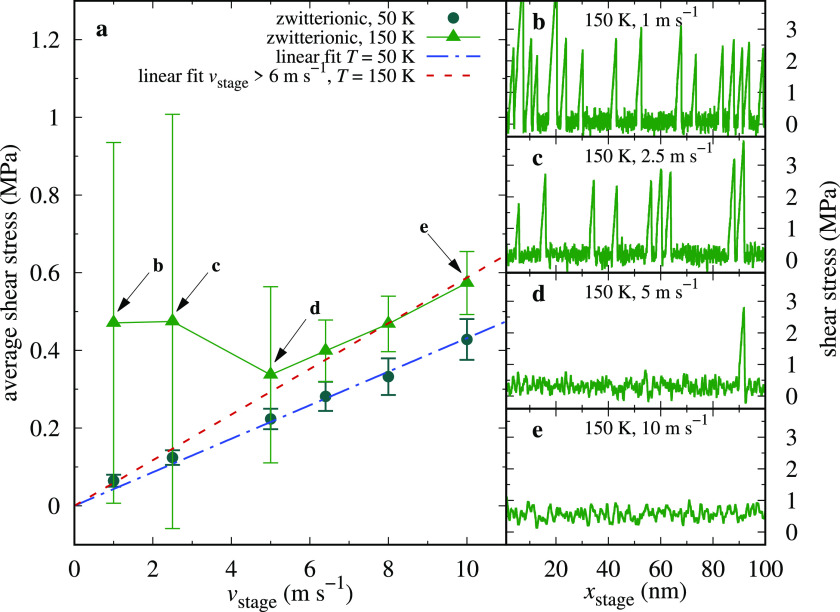
Stick–slip to smooth-sliding transition as a function of velocity. (a) The frictional shear stress as a function of the sliding velocity, zwitterionic system, *L* = 10 MPa. (b–e) Shear traces for the pointed velocities in panel (a) at 150 K. Dashed line: fit of *T* = 150 K and *v*_stage_ > 6 m·s^–1^. Dot-dashed line: fit of the *T* = 50 K, including all points.

Figure 2a displays the frictional shear stress as a function of temperature. Consider first the green symbols, reporting the simulations of the default model, the one involving zwitterionic head groups. At low temperature, a smooth-sliding regime (Figure 2c) characterized by extremely small friction is observed. In the smooth-sliding regime, the two layers remain substantially flat and well ordered due to the Coulombic interactions between cations and anions in the same layer (see Figures 3a and 4a): chains of opposite layers do not entangle, and they slide on top of each other encountering a quite small corrugation due to the discommensuration associated with the mutual angular misalignment. Starting from approximately *T* ≥ 200 K, stick–slip dynamics sets in (see Figure 2d), and friction increases substantially. As temperature is raised, thermal fluctuations promote out-of-plane chain movements leading to transient interlocking (see Figures 3b and 4b). The cationic chain head reaching through the opposite layer forms transient bonds with the anions belonging to two adjacent chains in the countersurface. The fraction of these bonds can be quantified through the “hooking fraction” *h*, i.e., the degree of interpenetration, defined quantitatively in the Supporting Information and reported in Figure 5a for the 300 K dynamics of Figure 2d. These bonds are responsible for the “stick” intervals, where the SUP remains essentially static and the driving spring elongates. The shear stress drops to nearly 0 after each slip and so does the hooking fraction (see Figure 3c and Figure 5a). The smooth-sliding and stick–slip motions of Figure 2c,d can be inspected in short movies reporting the last 6 ns of the simulations (i.e., the last 30 nm of the stage advancement in the shear traces), available as Movies S1 and S2. The transition from smooth sliding to stick–slip that we observe for increasing temperature depends on the sliding velocity and on the stiffness of the driving spring with smaller velocities and softer springs favoring stick–slip over smooth sliding.^18,46^ An important parameter of a tribological contact is the critical velocity *v*_c_ above which intermittent stick–slip dynamics tends to disappear. In our simulations, we can observe this disappearance as a function of *v*_stage_ at *T* = 150 K in Figure 6. With the adopted model parameters, our simulations show clear stick–slip dynamics for *v*_stage_ < 3 m·s^–1^ and smooth sliding for *v*_stage_ > 6 m·s^–1^. The model therefore predicts a critical velocity of ≃5 m·s^–1^. However, *v*_c_ can change by several orders of magnitude depending on system parameters. For example, at *T* = 50 K (see dot-dashed line in Figure 6a), the critical velocity is extremely small, and simulations capable of observing stick–slip would be far too long. It is therefore unfeasible to evaluate *v*_c_ systematically through simulations. Experimentally, in ref (47), this critical velocity is reported for SFA experiments involving squalane films. In those experiments, an increase of *v*_c_ with increasing temperature was observed: that result is compatible with the outcome of the present model.

Coming back to Figure 2a, as temperature increases to *T* ≃ 600 K, friction increases less and less until it peaks near 800 K. The transient bonds are numerous and relatively short-lived. The energy of each one of such bonds can be estimated (neglecting the small Morse contributions) by the difference in Coulomb attraction of a cation placed in between two adjacent anions of the opposite layer (distance ≃ 0.41 nm) and placed in its flat-layer configuration (distance ≃ 0.51 nm), which gives ≃85 meV. This transient bond energy is close to the thermal energy *k*_B_*T* ≃ 86 meV for *T* ≃ 1000 K, precisely in the temperature region of the observed friction peak. For even higher *T* > 1000 K, these bonds are destabilized and eventually friction decreases. In order to analyze the effect of the molecular charges on friction, we run analogous simulations for the charge-free system, reported as red curves in Figure 2. Remarkably, across the temperature range from *T* = 0 K to *T* = 550 K, friction is significantly larger than that for the zwitterionic system. The reason for this difference is that, even at 0 K, both molecular layers are significantly disordered due to the lack of long-range interactions. The chain-orientation disorder leads to a tilt-angle disorder too and to a significant corrugation of the two mutually sliding layers of the molecular heads (CA). This corrugation is also reflected in the consistently larger average SUP–SUB distance, shown in Figure 2b, compared to that in the zwitterionic case. Due to this extra corrugation, the hooked fraction of the charge-free system remains significant, even down to low temperatures.

The shear traces for the charge-free system (Figure 2e,f) exhibit stick–slip of smaller amplitude than those for the zwitterionic system. As illustrated in Figure 3d,e and in Movies S1, S2, S3, and S4, this is due to the charge-free molecules showing a larger density of protruding chains ready to interlock before the slip event has exhausted the energy stored in the pulling spring (Figure 3f). For *T* > 300 K, thermal fluctuations start to undermine the weaker Morse-type bonds of the protruding chains, leading to a progressive decrease in friction.

**Figure 7:**
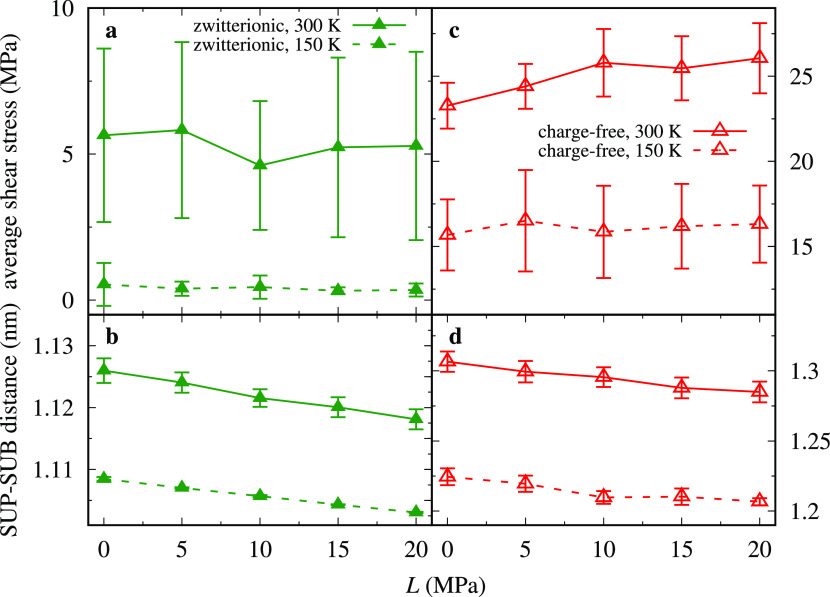
(a, c) Frictional shear stress and (b, d) the average distance between the rigid layers as a function of load *L* with *v*_stage_ = 5 m·s^–1^.

So far, the applied load *L* was fixed to 10 MPa. To explore how *L* affects the discussed phenomenology, we perform friction load cycles between 0 and 20 MPa and then back to 0 MPa as reported in Figure 7. The outcome of these simulations indicates that the shear stress does not change significantly upon loading, despite the chain-layer compression visible in Figure 7b,d. Additionally, the unloading data retrace those of the loading simulations with no visible hysteresis. For this reason, each point in Figure 7 is obtained as an average over both loading and unloading traces for that given load. The frictional shear traces for different loads are reported in Figures S2 and S3. The traces of the zwitterionic system, Figure S2, show that the critical velocity remains practically unchanged for all the investigated loads at *T* ≃ 150 K. Given such a weak friction dependence on load, it is essentially meaningless to define a friction coefficient for this model. From Figure 7, we expect the same conclusion at any *T* in the considered range. The charge-free reference system exhibits a marginally significant friction increase with load.

As for the velocity dependence, in the stick–slip regime, it is also expected to be very mild. Indeed, Figure S4 shows essentially no dependence as long as the dynamics is stick–slip. The zwitterionic system at *T* = 150 K reaches smooth sliding for *v*_stage_ > 6 m·s^–1^. The resulting friction linear increase, practically invisible in Figure S4, is clear in Figure 6a. Likewise, the smooth-sliding dynamics at 50 K also produces velocity-linear friction over all simulated velocities. In contrast, the charge-free system has stick–slip dynamics at all simulated temperatures resulting in velocity-independent friction.

Figure 5 and analogous plots for different dynamical conditions exhibit clear signs of correlation between the hooking fraction *h* and the frictional shear stress. We expect that *h* should also correlate with the total potential energy *U*. Specifically, we expect a decrease in total potential energy as the number of hooked stick points increases. This anticorrelation is illustrated in Figure S5 for the charge-free system at *T* = 0 K. However, at *T* = 300 K, the total potential energy is extremely noisy due to thermal fluctuations (see Figure 5), and these anticorrelations are hard to detect visually.

**Figure 8:**
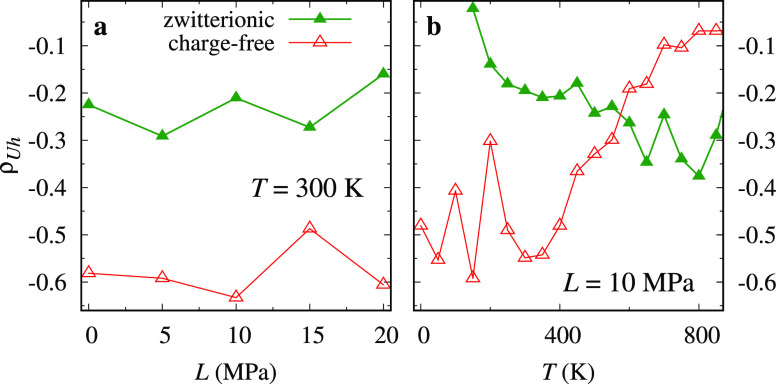
Correlation coefficient ρ_*Uh*_ between the hooked fraction and total potential energy, eq 2, for the zwitterionic and charge-free systems at *v*_stage_ = 5 m·s^–1^ as a function of (a) the applied load and (b) the temperature. As in smooth sliding *h* ≡ 0, ρ_*Uh*_ is defined for stick–slip dynamics only.

Figure S6 illustrates these correlations with scatter plots for the zwitterionic (panel a) and charge-free (panel b) models at *T* = 300 K, related to the traces of Figure 2d,f. These scatter plots provide qualitative hints of these correlations. For a quantitative evaluation of these correlations, we calculate the Pearson correlation coefficient^48^
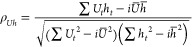
2and report it as a function of load and temperature in Figure 8. ρ_*Uh*_ is systematically negative, confirming the expected anticorrelation. At *T* = 300 K, the charge-free model exhibits more negative anticorrelation compared to the zwitterionic model at all the investigated loads (Figure 8a). As a function of temperature, so far, the zwitterionic and charge-free models behave quite differently. The zwitterionic model has null *h* at low temperatures (smooth sliding), and therefore, ρ_*Uh*_ is undefined. As stick–slip develops, ρ_*Uh*_ becomes more and more negative. In contrast, the charge-free system exhibits stick–slip down to a temperature of zero with the correspondingly largely negative ρ_*Uh*_. As temperature is raised, these correlations approach zero and correspondingly friction decreases (Figure 2a).

## Discussion and Conclusions

In this work, we have introduced and studied a model that offers a microscopic implementation for friction mediated by thermally activated formation and rupture of interfacial contacts, a subject which so far has been explored by means of phenomenologic theory.^12,49−56^ This kind of theory has been found to have wide applications in describing friction and wear in dry and lubricated contacts over a broad range of lengths and revealed the origin of new, unexpected phenomena such as non-Amonton’s variation of friction force with normal load and nonmonotonic dependence of friction on sliding speed and temperature. The microscopic model proposed here advances understanding mechanisms underlying the phenomenological theory and offers new pathways for the rational control of frictional response.

The main outcome of this model consists of a remarkable increase in friction with temperature. Depending on the operating conditions, sliding can occur with stick–slip or smooth advancement. The high-friction stick state results from the interlocking of molecular chains promoted by thermal fluctuations. In the proposed model, Coulombic interactions between the zwitterionic macromolecules enhance the intermolecular interactions, favoring flat and ordered layers and smooth sliding at low temperatures. We can imagine different types of intermacromolecule interactions, for example, hydrogen bonding or solvent-mediated couplings, that could support a similar kind of low-temperature ordering. Thermally activated interlocking is therefore also likely to account for thermally enhanced friction in more general contexts, as for example, in the experiments of ref (47).

Zwitterionic macromolecules are promising systems for the control of friction by externally applied electric fields. The change in the orientation of the molecular zwitterionic heads due to the field may affect interlocking and, therefore, friction.^57^ We hope that this model stimulates further experiments in this and related directions.

## Supporting Information Available

Details of the chain arrangement, supercell geometry, and intermolecular interactions; the average shear stress as a function of the damping parameter γ (Figure S1); the definition of the hooking fraction *h*; the frictional shear traces of the zwitterionic (Figure S2) and charge-free (Figure S3) systems for the load dependence of Figure 7a,c; shear stress and SUP–SUB distance dependence on the sliding velocity for zwitterionic and charge-free systems (Figure S4); correlation of the hooking fraction, shear stress, and total potential energy for the charge-free system at *T* = 0 K (Figure S5); scatter plots illustrating the anticorrelation between the total potential energy and the hooking fraction that determine the *T* = 300 K points in Figure 8b for zwitterionic and charge-free systems (Figure S6); technical details about 4 short supporting movies illustrating the MD simulations corresponding to the last 30 nm of the stage’s displacement in the shear traces of Figure 2c–f (PDF)
Last 6 ns of the MD simulation corresponding to the force trace shown in Figure 2c (MP4)
Last 6 ns of the MD simulation corresponding to the force trace shown in Figure 2d (MP4)
Last 6 ns of the MD simulation corresponding to the force trace shown in Figure 2e (MP4)
Last 6 ns of the MD simulation corresponding to the force trace shown in Figure 2f (MP4)
