# Supplementary material for: Thermal Friction Enhancement in Zwitterionic Monolayers
Source: J Phys Chem C Nanomater Interfaces. 2022 Feb 1;126(5):2797–805. doi: 10.1021/acs.jpcc.1c09542 (PMC8842320; doi:10.1021/acs.jpcc.1c09542)
Supplement: Supplementary file 1 — jp1c09542_si_001.pdf [file jp1c09542_si_001.pdf]

Supporting Information for Thermal Friction Enhancement in Zwitterionic Monolayers

Melisa M. Gianetti,[*,†] Roberto Guerra,[‡] Andrea Vanossi,[¶,§] Michael Urbakh,[‖] and Nicola Manini[†]

†*Dipartimento di Fisica, Università degli Studi di Milano, Via Celoria 16, Milano, 20133 Italy*

‡*Center for Complexity and Biosystems, Department of Physics, University of Milan, via Celoria 16, Milano, 20133, Italy*

¶*CNR-IOM, Consiglio Nazionale delle Ricerche - Istituto Officina dei Materiali, c/o SISSA, Via Bonomea 265, 34136 Trieste, Italy*

§*International School for Advanced Studies (SISSA), Via Bonomea 265, 34136 Trieste, Italy*

‖*Department of Physical Chemistry, School of Chemistry, The Raymond and Beverly Sackler Faculty of Exact Sciences and The Sackler Center for Computational Molecular and Materials Science, Tel Aviv University, Tel Aviv 6997801, Israel*

E-mail: melisamariel@gmail.com

Supercell Geometry

Intending to arrange periodically two arrays of molecules according to triangular lattices we introduce primitive vectors

$$\mathbf{a}_1 = a\,(\cos(\phi/2), \sin(\phi/2)) \qquad \mathbf{a}_2 = a\,(\cos(\pi/3+\phi/2), \sin(\pi/3+\phi/2)) \tag{S1}$$

$$\mathbf{b}_1 = a\,(\cos(-\phi/2), \sin(-\phi/2)) \qquad \mathbf{b}_2 = a\,(\cos(\pi/3-\phi/2), \sin(\pi/3-\phi/2)) \tag{S2}$$

with spacing $a = 0.82$ nm, and rotated by $\phi/2$ in opposite directions.

We need a common periodicity and therefore, a matching lattice vector

$$m_1\mathbf{a}_1 + m_2\mathbf{a}_2 = m_2\mathbf{b}_1 + m_1\mathbf{b}_2 \tag{S3}$$

with integers m_1 and m_2. This evaluation of this common lattice vector is a special case of the theory described in Ref. 1.

We find that a reasonably-sized supercell is obtained with $m_1 = 13$ and $m_2 = 7$. We solve, e.g., the y component of Eq. (S3):

$$m_1\sin(\phi/2) + m_2\sin(\pi/3+\phi/2) = m_1\sin(\pi/3-\phi/2) - m_2\sin(\phi/2). \tag{S4}$$

We obtain

$$\phi = 2\arctan\left(\frac{\sqrt{3}}{10}\right) \simeq 19.65286^\circ. \tag{S5}$$

With the adopted geometry both rotated lattices have lattice points along x and y. To determine them we solve the null components of the equations

$$n_1\mathbf{a}_1 + n_2\mathbf{a}_2 = (l_x, 0) \tag{S6}$$

$$n_1'\mathbf{a}_1 + n_2'\mathbf{a}_2 = (0, l_y), \tag{S7}$$

obtaining $n_1 = 11$, $n_2 = -2$, $n'_1 = -7$ and $n'_2 = 20$. The remaining components of the equations provide

$$l_x = a\left[n_1 \cos(\phi/2) + n_2 \cos(\phi/2 + \pi/3)\right] \simeq 8.32 \text{ nm} \tag{S8}$$

$$l_y = a\left[n'_1 \sin(\phi/2) + n'_2 \sin(\phi/2 + \pi/3)\right] \simeq 14.41 \text{ nm.} \tag{S9}$$

Alternatively, the opposite corner (l_x, l_y) of a rectangular supercell with a corner at the origin $(0, 0)$ is obtained as $(l_x, l_y) = 4\mathbf{a}_1 + 18\mathbf{a}_2$.

Cutoff of the Two-Body Potential

For the non-bonded pairwise particle-particle interactions we adopt a Morse potential with a standard shift and a linear term added as follows:

$$V(r) = \begin{cases} V_{\text{Morse}}(r) - V_{\text{Morse}}(R_c) - (r - R_c) \left.\frac{dV_{\text{Morse}}}{dr}\right|_{r=R_c}, & r < R_c \\ 0, & r \geq R_c \end{cases} \tag{S10}$$

so that the truncated potential vanishes smoothly at R_c.

Long-Range Solver for Coulomb Interactions

The PPPM solver used for systems, such as ours, which are periodic in x and y, but not in z, requires an ad-hoc extension. The system is treated as if it was periodic in z, but inserting an empty volume between the slabs and thus removing unphysical dipole inter-slab interactions. For the parameter setting the fraction of empty volume in between slab repetitions, we adopt the value 3.0 recommended by the developers of the simulation software LAMMPS.[2] We explicitly verified that, by improving the accuracy of the PPPM solver beyond 10^{-4} eV/nm, neither quantitative effects on the sliding friction nor qualitative effects on the system dynamics are detectable.

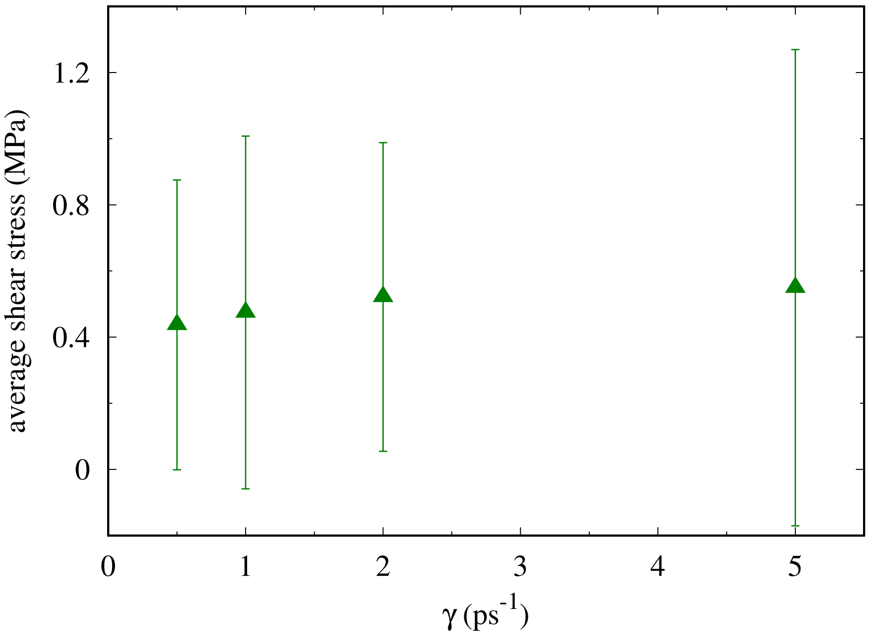

Figure S1: The average shear stress as a function of the damping parameter γ for the zwitterionic model. Simulations are carried out for $v_{stage} = 2.5\,\mathrm{m{\cdot}s^{-1}}$, $T = 150\,\mathrm{K}$, $L = 10\,\mathrm{MPa}$.

The Hooking Fraction h

In order to quantify the degree of interpenetration of the chains we introduce a "hooking fraction" h as the fractional number of chains whose cation crosses the average level of cations of the opposite layer, like the highlighted chains in Fig. 4b,d of the main text. The definition for h is the following:

$$h = \frac{1}{2}\left(h_{\mathrm{SUP}} + h_{\mathrm{SUB}}\right), \tag{S11}$$

where

$$h_{\mathrm{SUB}} = \frac{1}{N_{\mathrm{SUB}}} \sum_{j=1}^{N_{\mathrm{SUB}}} \theta(z_j^{\mathrm{CA}} - \bar{z}_{\mathrm{SUP}}) \tag{S12}$$

$$h_{\mathrm{SUP}} = \frac{1}{N_{\mathrm{SUP}}} \sum_{i=1}^{N_{\mathrm{SUP}}} \theta(\bar{z}_{\mathrm{SUB}} - z_i^{\mathrm{CA}}). \tag{S13}$$

Here $\theta()$ is the usual θ function, equal to one or zero according to the sign of its argument, and

$$\bar{z}_{\mathrm{SUP}} = \frac{1}{N_{\mathrm{SUP}}} \sum_{i=1}^{N_{\mathrm{SUP}}} z_i^{\mathrm{CA}}, \qquad \bar{z}_{\mathrm{SUB}} = \frac{1}{N_{\mathrm{SUB}}} \sum_{j=1}^{N_{\mathrm{SUB}}} z_j^{\mathrm{CA}}. \tag{S14}$$

For example, the hooking fraction as a function of time is illustrated in Fig. 5a. h clearly correlates with the stick-slip dynamics.

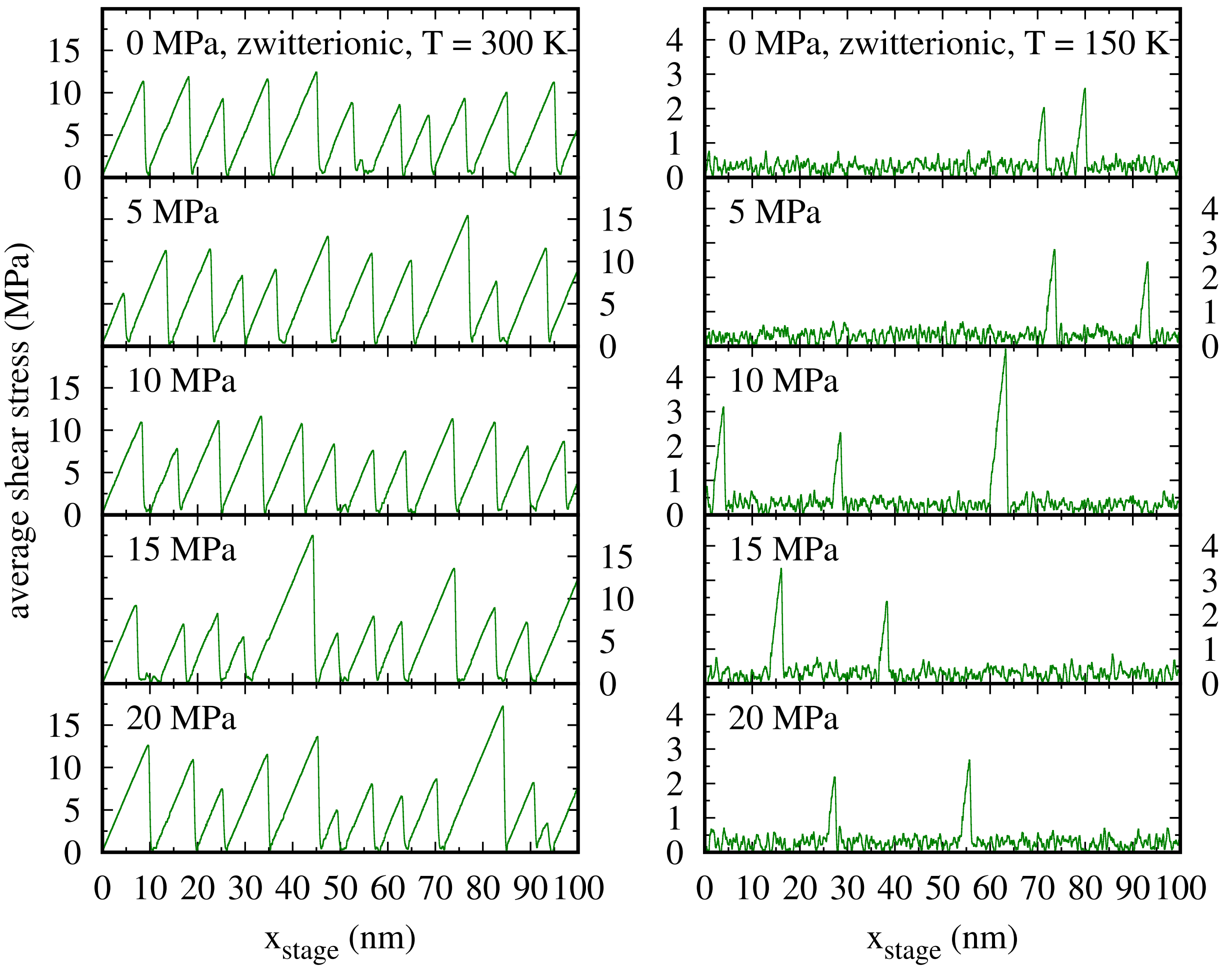

Figure S2: The frictional shear traces of the zwitterionic model obtained for stepwise increasing load L. Averages (including the load-decreasing traces – not shown) are reported in Fig. 7a of the main text. Simulation conditions: $v_{stage} = 5\,\mathrm{m{\cdot}s^{-1}}$, (left) $T = 300\,\mathrm{K}$ and (right) $T = 150\,\mathrm{K}$.

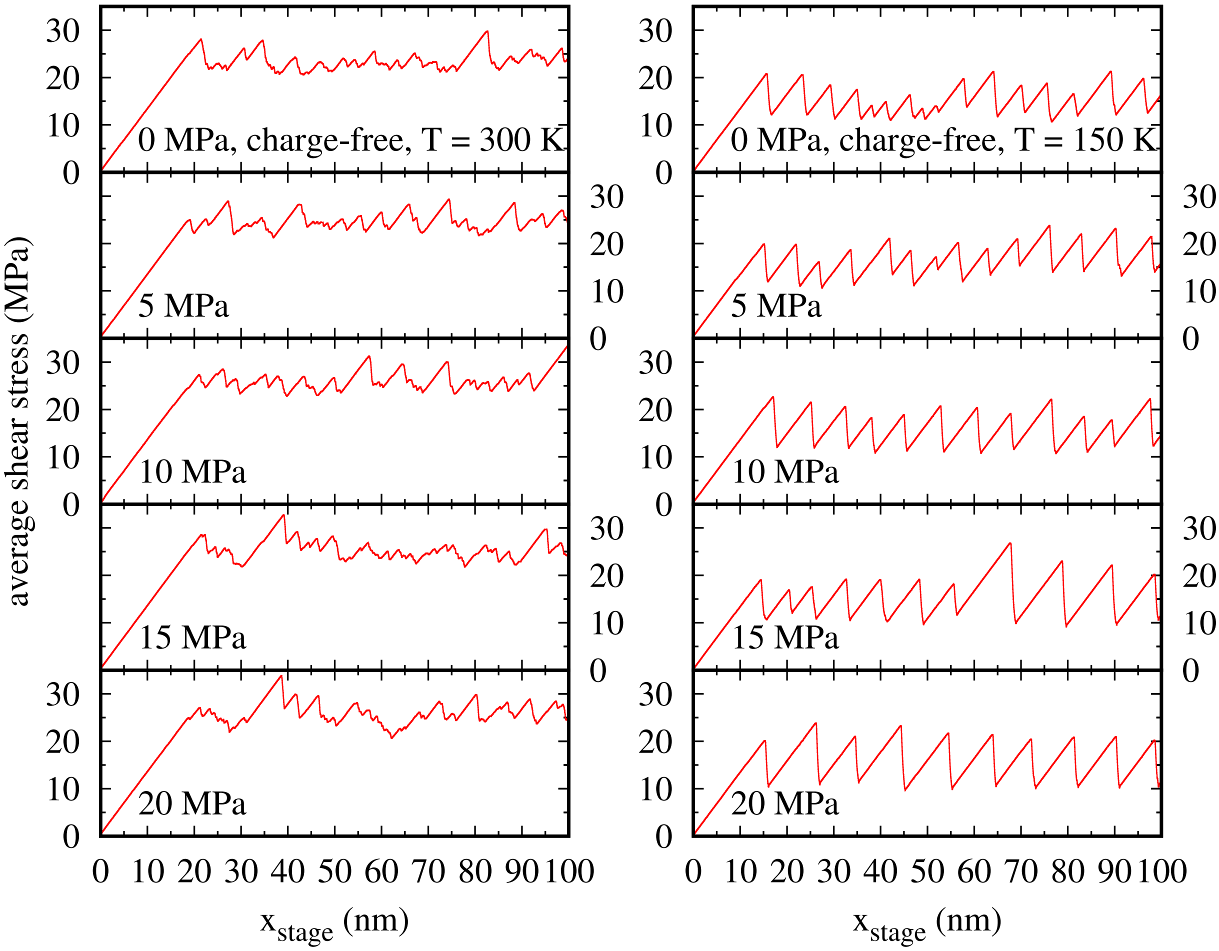

Figure S3: Same as Fig. S2 but for the charge-free model – Averages reported in Fig. 7c of the main text.

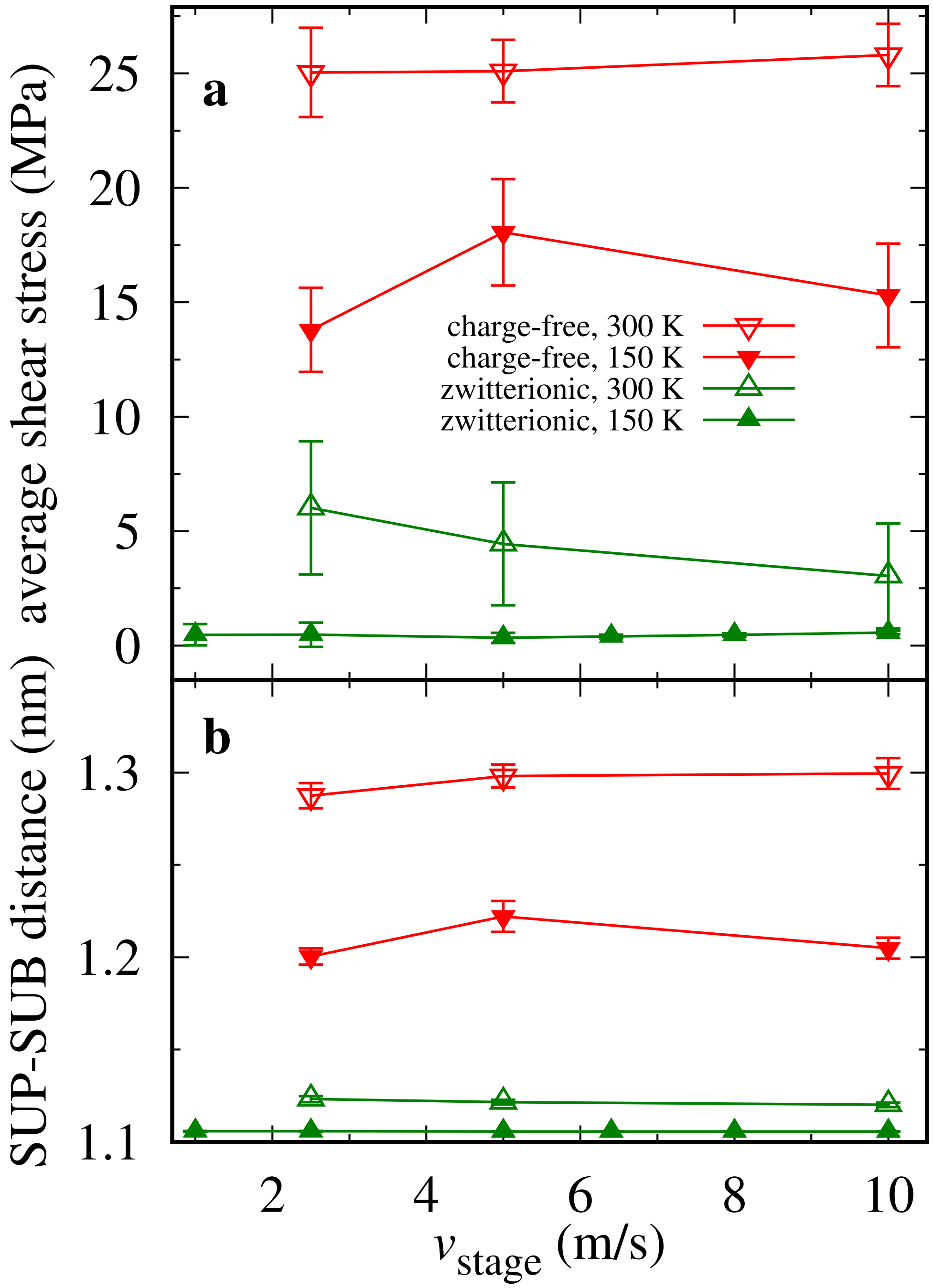

Figure S4: (a) The frictional shear stress and (b) the distance between the rigid layers as a function of the advancement velocity of the stage for the two indicated temperatures and for load $L = 10\,\text{MPa}$.

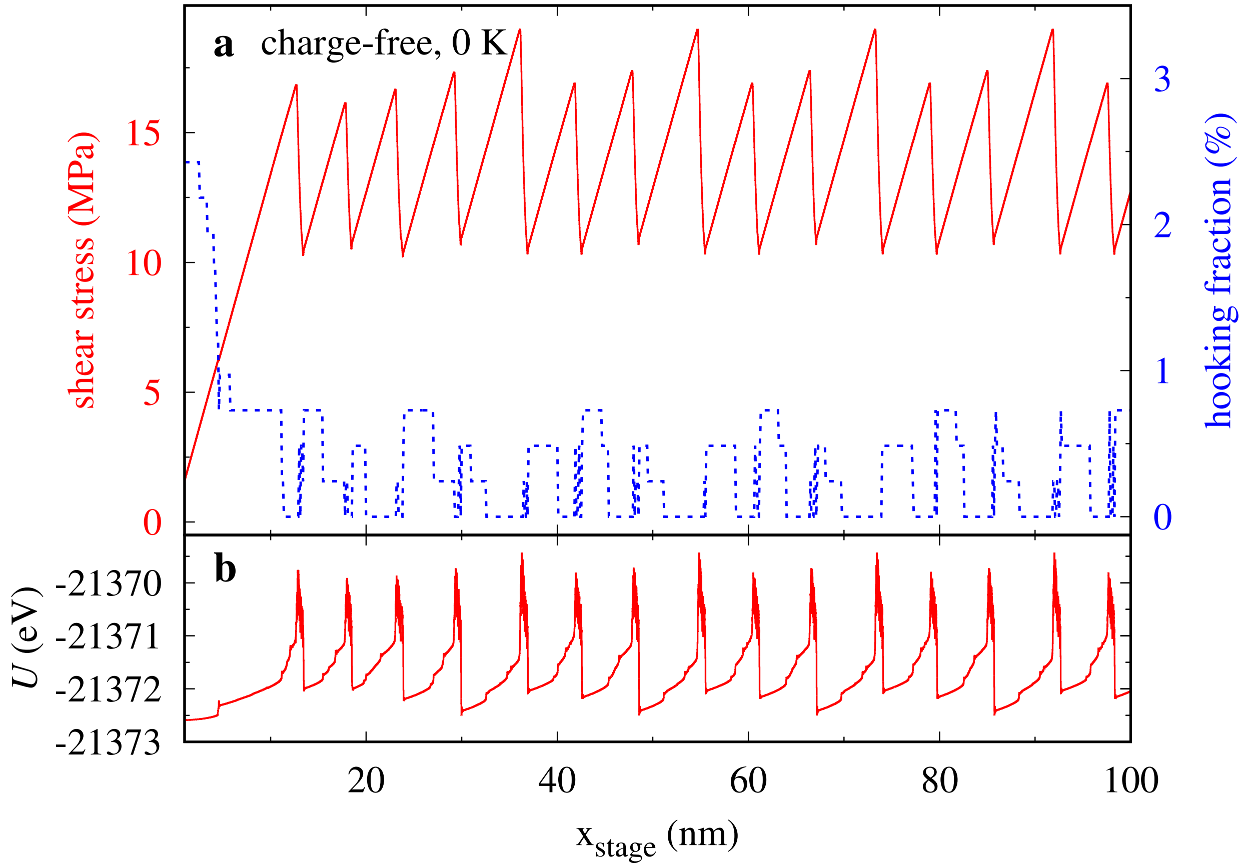

Figure S5: (a) The percentile hooking fraction h as a function of the stage displacement correlated with the frictional shear stress for the charge-free model at $T = 0$ K. (b) The total potential energy for the same simulation.

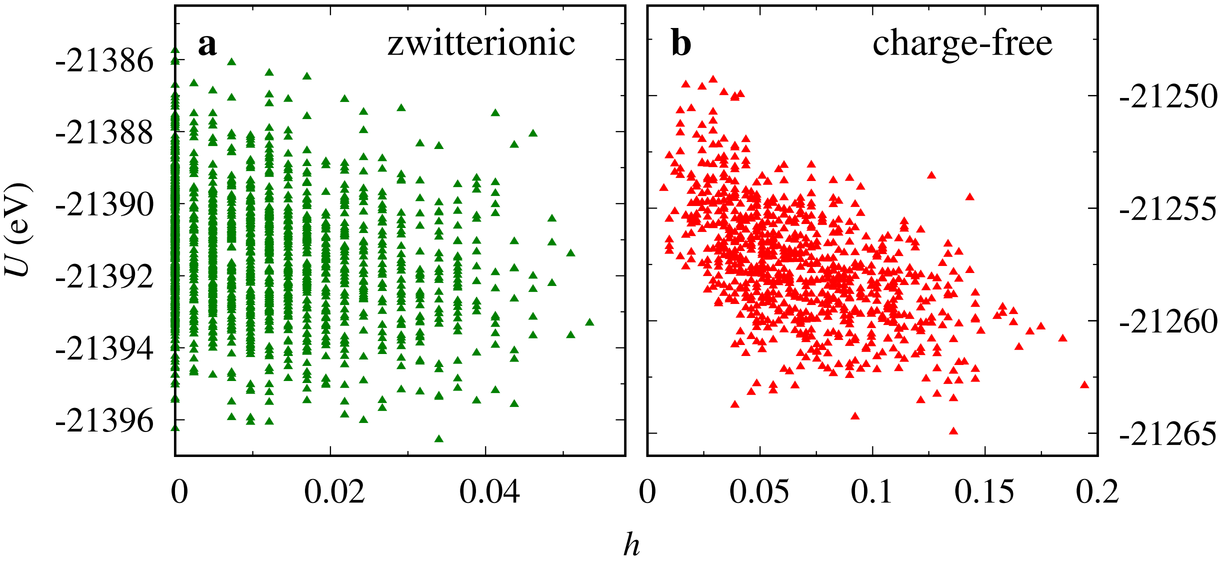

Figure S6: Scatter plot illustrating the correlation between the total potential energy and the hooked fraction for (a) zwitterionic system (b) charge-free system. Correlation coefficients for these data are reported in Figure 8b of the paper. $L = 10$ MPa and $T = 300$ K.

SI Movies

Each of the SI movies reports the final 6 ns (i.e. the last 30 nm displacement) of a MD simulation. In simulation time, the frame rate is 1 frame every 20 ps. In running time, the frame rate is 10 frames per second. For clarity, like in Fig. 3 of the main text, the movies only include a 5 nm y-thick slice of the simulation cell (whose entire y-side is 14.41 nm).

Each movie contains one highlighted SUP particle to make the displacement of the rigid top layer more evident.

- **zwitterionic_150K.mp4**: the last 6 ns of the MD simulation corresponding to the force trace shown in Figure 2c;
- **zwitterionic_300K.mp4**: the last 6 ns of the MD simulation corresponding to the force trace shown in Figure 2d;
- **charge-free_150K.mp4**: the last 6 ns of the MD simulation corresponding to the force trace shown in Figure 2e;
- **charge-free_300K.mp4**: the last 6 ns of the MD simulation corresponding to the force trace shown in Figure 2f.

References

(1) Grey, F.; Bohr, J. A symmetry principle for epitaxial rotation. *Europhys. Lett.* **1992**, *18*, 717.

(2) Yeh, I.-C.; Berkowitz, M. L. Ewald summation for systems with slab geometry. *J. Chem. Phys.* **1999**, *111*, 3155–3162.
